# Safety outcomes of mucomuscular closure versus conventional clip closure in ESD of large (> 15 mm) nonpedunculated colorectal polyps (LNPCPs)

**DOI:** 10.1007/s10151-025-03261-w

**Published:** 2025-12-24

**Authors:** T.‐Y. Chen, L.-F. Wu, X.-Y. Xu, Y.-B. Liu, Y.-F. Zhang, W.‐F. Chen, Q.‐L. Li, J.‐W. Hu, J.-X. Xu, J. Cheng, K.-Q. Zhou, P.-H. Zhou, Y.‐Q. Zhang

**Affiliations:** 1 Department of Endoscopy Center and Endoscopy Research Institute, Zhongshan Hospital, Fudan University, 180 Fenglin Road, Shanghai, 200032 China; 2 Department of Endoscopy, Shanghai Collaborative Innovation Center, Shanghai, China

**Keywords:** C-ESD, Mucomuscular closure, PEECS

## Abstract

**Background and aims:**

Post-endoscopic submucosal dissection (ESD) electrocoagulation syndrome (PEECS) is a recognized limitation of colorectal ESD (C-ESD) associated with morbidity, additional costs, and prolonged admission. Reliable closure of C-ESD defects can decrease the incidence of PEECS. We introduce a novel mucomuscular closure technique that involves direct closure of the muscularis propria using through-the-scope clips (TTSC). We evaluate the feasibility and efficacy of the modified closure technique in prevention of post-C-ESD PEECS.

**Methods:**

We conducted a prospective cohort study of consecutive C-ESDs at a single tertiary center between January 2017 and October 2023. Patients who underwent C-ESD with mucomuscular closure or conventional closure with TTSC were enrolled. The primary outcome was the incidence and clinical outcome of PEECS. Secondary outcomes were rates of complete defect closure and severe adverse events (SAEs).

**Results:**

A total of 764 patients were included in this study. The incidence of PEECS was significantly lower in the mucomuscular closure group versus conventional closure group (2.5% versus 15.0%, *P* < 0.001). No SAEs occurred in mucomuscular closure group, whereas two patients had delayed perforation, and two had delayed bleeding in the conventional closure group. In mucomuscular closure group, there was no difference in PEECS occurrence between complete closure (5/218, 2.3%) and partial closure (3/105, 2.9%). No TTSC-related perforation occurred in the process of defect closure.

**Conclusions:**

Mucomuscular closure with TTSC in C-ESDs is effective in preventing PEECS and other postoperative complications.

**Supplementary Information:**

The online version contains supplementary material available at 10.1007/s10151-025-03261-w.

## Introduction

Colorectal endoscopic submucosal dissection (C-ESD) is widely accepted for the effective removal of large colorectal superficial neoplasms. ESD allows en bloc resection regardless of lesion size and location, facilitates accurate histopathological assessment, and achieves a lower recurrence rate compared to endoscopic mucosal resection (EMR) [1].

Although C-ESD has several advantages, it is technically challenging due to the thin intestinal wall and limited luminal space. Post-ESD electrocoagulation syndrome (PEECS) is a common adverse event, with incidence ranging between 6.8% and 40.1% [2–5]. PEECS develops due to transmural injury from electrocauterization without perforation and serosal inflammation resulting in localized peritonitis [6]. Exposure of the defect to bacterial infection may be an additional mechanism. [7–9] Previous studies reported that reliable closure of ESD defects can decrease PEECS [10, 11]. There is also few literature that suggest metal clip closure cannot reduce the occurrence of adverse events including PEECS [12]. Conventional clip closure of large defects can be challenging and may achieve apposition of mucosal layer only, leaving a dead space between the mucosa and muscularis propria (MP) layer. This dead space can lead to the premature detachment of clips and subsequent exposure of the defect.

To overcome this limitation, we introduce a novel closure technique adapted from seromuscular suture techniques applied in surgical closure of intestinal wall defects, termed the modified mucomuscular closure method. This method involves direct closure of the MP layer using through-the-scope-clips (TTSC), which effectively reduces tension from surrounding tissues and eliminates dead space. Our study aims to evaluate the effect of the modified closure technique on the incidence of PEECS as well as its feasibility and safety.

## Methods

### Study design

**Fig. 1:**
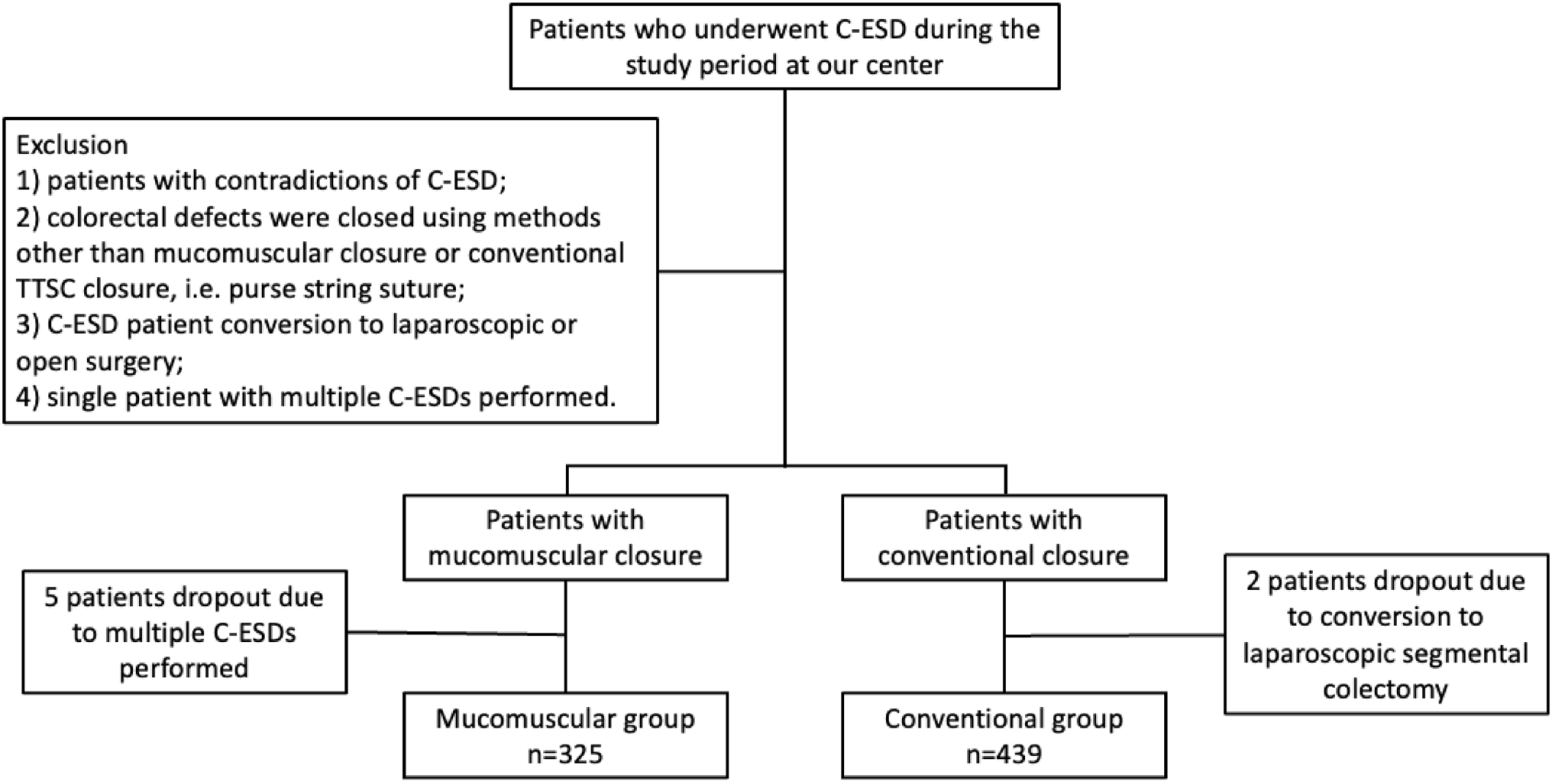
Flow chart of the study

The mucomuscular closure cohort included and prospectively followed consecutive patients with large (> 15 mm) nonpedunculated colorectal polyps (LNPCPs) who underwent C-ESD with mucomuscular closure at the Endoscopy Center of Zhongshan Hospital, Fudan University between August 2017 and October 2023. The conventional closure cohort consisted of retrospective cases of patients with C-ESD with large LNPCPs between January 2017 and August 2023. The indications for colonic ESD were lesions not suitable for removal by en bloc EMR due to size, fibrosis, or suspicion of superficial carcinoma [13]. Exclusion criteria were: (1) patients with contradictions of C-ESD, such as advanced colorectal cancer, severe cardiovascular diseases, untreated coagulation disorders, and so on; (2) colorectal defects were closed using methods other than modified closure technique and conventional TTSC closure, i.e., purse string closure; (3) patients with C-ESD requiring conversion to laparoscopic or open surgery; (4) single patient with multiple C-ESDs performed (Fig. 1).

This study was performed in accordance with the 2008 revision of the Declaration of Helsinki. The Ethics Committee of Zhongshan Hospital approved this study (no. B2021-692), and all patients provided written informed consent.

### C-ESD procedures

C-ESD procedures were performed by expert endoscopists at the Endoscopy Center of Zhongshan Hospital, Fudan University. Procedures were completed under propofol sedation or general anesthesia if serious interference from respiration occurred. An ERBE VIO-200D electrocautery unit (ERBE Electromedizin, Tübingen, Germany) was used for electrosurgery. Endocut mode was applied with effect 2–3 (output limit 50 W), cut distance 2–3, and cut interval 3–4 depending on the size of the lesion and endoscopist’s preference. ESDs were performed with a standard single accessory-channel colonoscope (PCF-260 J, Olympus, Japan) and gastroscope (GIF-Q260J, Olympus, Japan) for colonic and rectal LNPCPs respectively. A transparent cap (D-201–11802, D-201–12704 Olympus, Japan) was applied. Submucosal injection with a mixture of glycerin fructose and indigo carmine was performed. Then, a circumferential incision was made around the lesion with an ESD knife (hook knife, KD-620QR, Olympus, Japan; dual knife, KD-650Q, Olympus, Japan; water-jet hybrid knife, ERBE, Germany). The incision created a mucosal flap and access for submucosal dissection. The additional submucosal solution was injected under the incised mucosal flap to allow electrocautery dissection until the entire lesion was released from its submucosal attachment. Visible vessels in the resected area were coagulated to prevent delayed bleeding. The mucosal layer or the MP layer was then closed with metal clips. All lesions were subjected to pathological examination.

Patients were categorized into two groups based on the defect closure method used: the conventional closure group and the mucomuscular closure group.

### Conventional closure

The conventional closure group was composed of patients who underwent C-ESD followed by closure of the mucosal defect edges with TTSC. It is important to note that this conventional closure technique was consistently employed, independent of the extent of involvement of the submucosal or muscularis propria layers.

### Mucomuscular closure

**Fig. 2:**
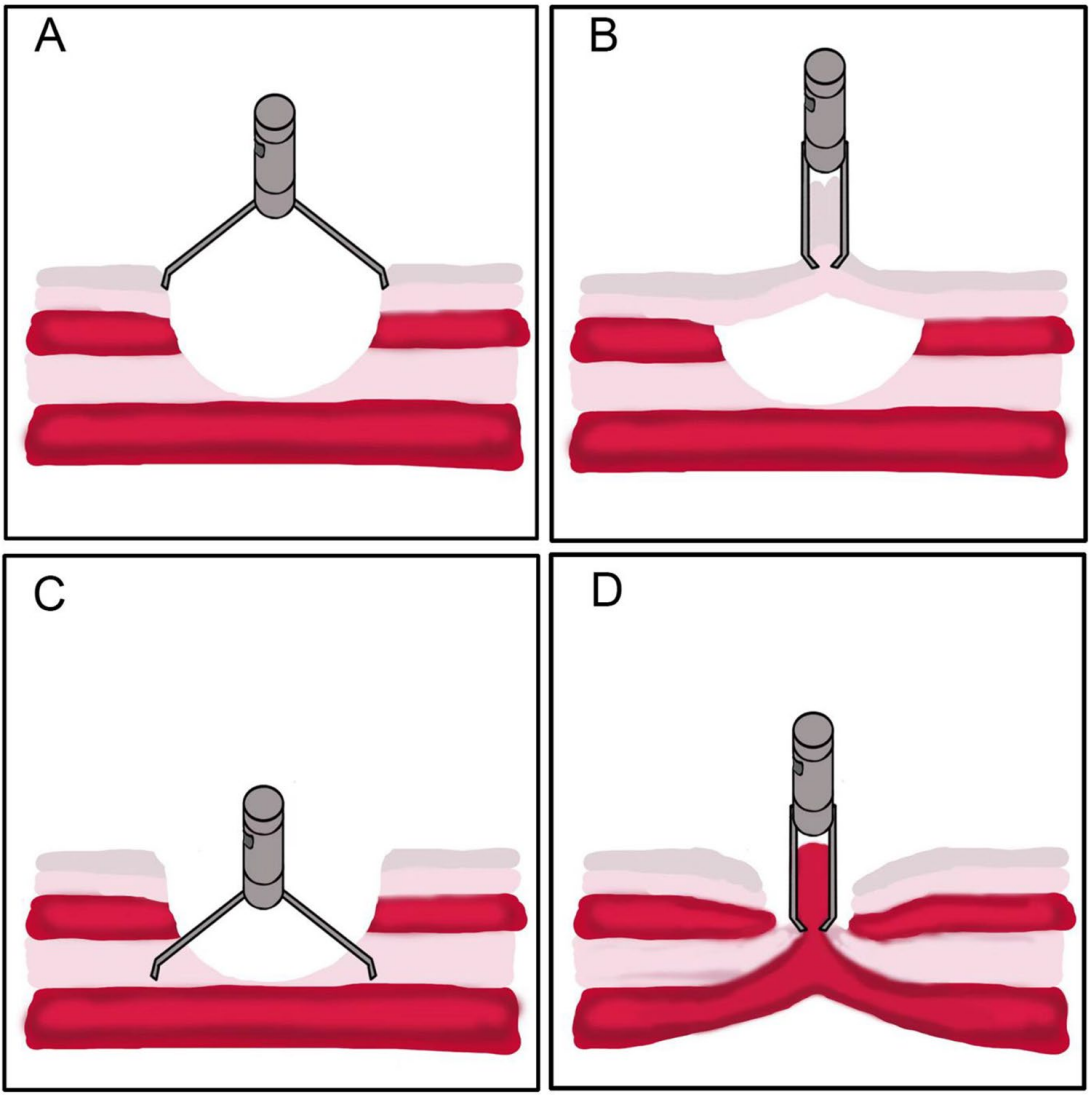
Demonstration of mucomuscular closure of C-ESD. A schematic diagram of through-the-scope-clip closure methods. **A**, **B** Conventional closure method may leave a dead space between mucosal layer and MP layer. **C**, **D** Mucomuscular closure method ensures muscularis propria apposition, eliminates dead space and provide reliable support of intestinal integrity

**Fig. 3:**
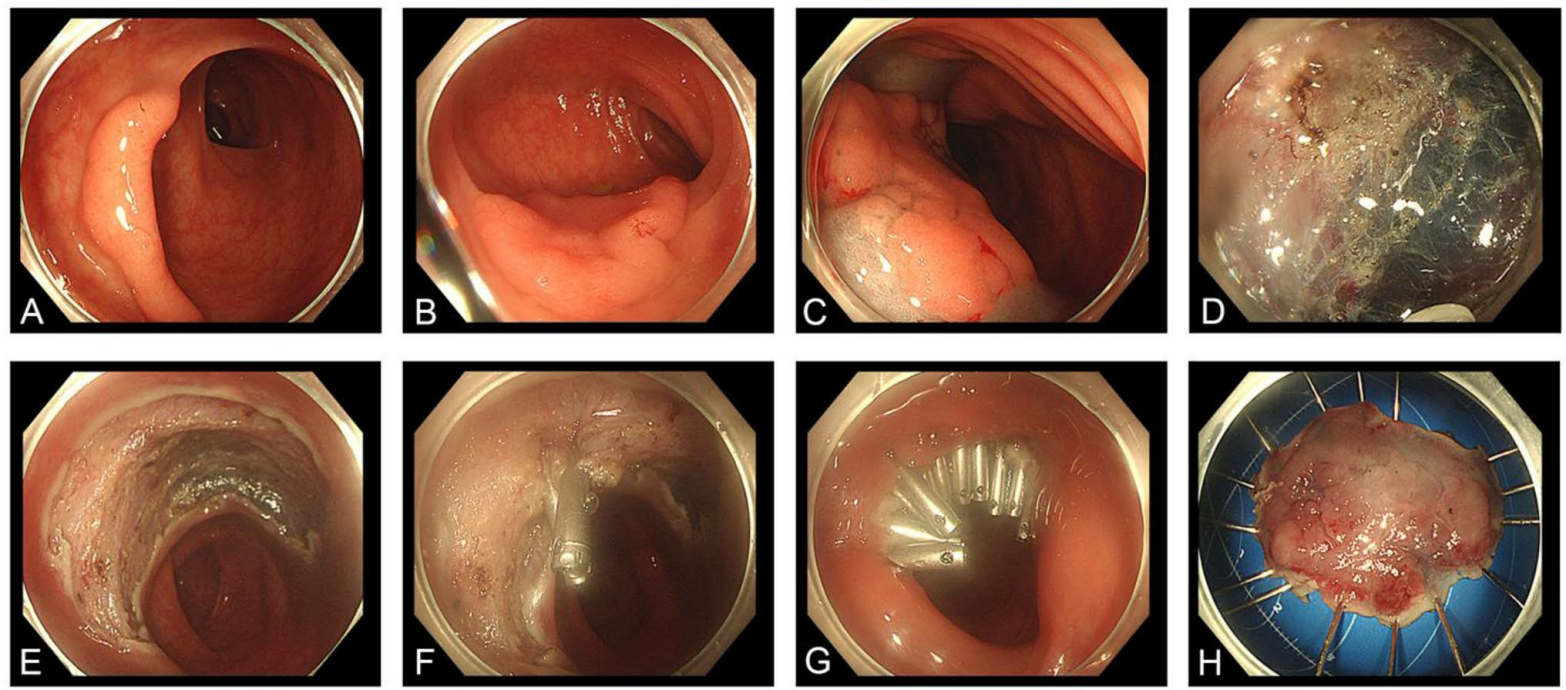
Colonic endoscopic mucosal dissection using mucomuscular closure method. **A**, **B**. The endoscopic view showed a 2.5 cm laterally spreading tumor (LST). **C** Adequate submucosal injection using glycerin-fructose suggested the lesion was well lifted. **D** The lesion was dissected from surrounding tissues. **E** The large defect of the colon wall after tumor resection. **F**, **G** Metal clips were used to directly suture the muscularis propria layer to close the wound. **H** Macroscopic view of the specimen. Final pathologic finding was high grade neoplasia

The mucomuscular closure group included patients who were subjected to the mucomuscular suturing method. The mucomuscular closure technique focuses on reducing the shear tension of the intestinal wall and preventing minute leakage of the MP layer regardless of the mucosal layer alignment. As the TTSC approaches the MP layer, air is suctioned to reduce the tension of the intestinal wall and allow TTSC to grasp the inverted MP layer easily. The above steps are repeated until reliable closure of the exposed MP layer is achieved (Figs. 2, 3, Video 1). In cases where injury to the MP layer occurs during C-ESD, mucomuscular closure becomes essential to mitigate the risk of delayed perforation and other serious adverse events. Following mucomuscular closure of the defect, the mucosal layer may remain partially open. However, as long as the muscularis propria has been adequately sutured, complete mucosal closure is not necessary. The mucomuscular closure was performed by three highly skilled endoscopists who have experience of over 500 C-ESD cases.

### Data and outcomes

Details on patient demographic, lesion characteristics, (age, sex, lesion size and site, number of lesions, and preoperative test results), and procedure outcomes (operation time, complete closure, adverse events, length of postoperative hospital stay, relevant exams, treatments, and pathological findings) were recorded. Fulfillment of discharge criteria was defined as the absence of fever, clinical evidence of peritonitis, or other procedure-related symptoms following a minimum 24 h observation period. Routine telephone follow-up was performed at 12 weeks. Adverse events included PEECS, delayed bleeding, delayed perforation, and other SAEs that require medication or interventional treatment.

Complete closure was defined as absence of exposed defect after clip closure. PEECS was defined as fever (> 37.8 °C), abdominal pain and/or tenderness, and/or leukocytosis (> 10 × 10^9^/L),) without definitive evidence of perforation occurring 12 h or later after C-ESD. In suspected PEECS cases, an abdominal CT scan was obtained to exclude delayed perforation. Delayed perforation was defined as radiologically confirmed perforation with the presence of extra-luminal free air. Delayed bleeding was defined as bleeding that required endoscopic hemostasis or readmission within 14 days after C-ESD.

The primary outcome of our study was the incidence of PEECS. Secondary outcomes were complete closure rate and SAEs.

### Statistical analysis

All the statistical analyses were conducted using the Statistical Package for Social Sciences (SPSS) software (version 25, SPSS, Inc., Chicago, IL, USA). Results were expressed as mean ± standard deviation or percentage. Continuous variables were compared between the two groups and analyzed using the unpaired *t*-test. Categorical variables were compared between the two groups using the chi-squared test or Fisher’s exact test. The odds ratio (OR) and 95% confidence interval (CI) were calculated using logistic regression analysis to evaluate the predictors of PEECS. Variables with *P* < 0.1 on univariate were added to the multivariate logistic regression model to identify independent risk factors for PEECS. A *P* value < 0.05 was considered statistically significant, and a *P* value < 0.1 was considered to be a statistical trend.

## Results

A total of 764 patients who underwent C-ESD between January 2017 and October 2023 were included with 439 in the conventional closure group and 325 in mucomuscular closure group. Patient and lesion characteristics are summarized in Table 1. The mean age of patients was 63.0 ± 10.9 years (range 28–93 years) and 56.5% were male. The mean size of the colonic lesions was 31.3 ± 13.0 mm (range 12.0–100.0 mm). The mean operation time for C-ESD was 55.3 ± 33.2 min (range 10.0–300.0 min) (Table 2).

**Table 1:** Clinical characteristics of patients

Characteristics		*P* value
Age, median (range), years	63 (28–93)	
Sex		
Male	432 (56.5%)	
Female	332 (43.5%)	
Type of closure		
Mucomuscular	325 (42.5%)	
Conventional	439 (57.5%)	
Operation time, median (range), min	55.3 (10.0–300.0)	0.261
Mucomuscular	53.7 ± 32.7	
Conventional	56.5 ± 33.5	
Tumor size, median (range), mm	31.3 (12.0–100.0)	0.204
Mucomuscular	30.6 ± 11.8	
Conventional	31.8 ± 13.8	
Pathological diagnosis		
Low-grade neoplasia	269 (35.2%)	
High-grade neoplasia	301 (39.4%)	
Carcinoma	146 (19.1%)	
Other	48 (6.3%)	
PEECS, percent (*n*)		< 0.001
Mucomuscular	2.5% (8/325)	
Conventional	15.0% (66/439)	
Delayed perforation		0.510
Mucomuscular	0	
Conventional	0.5% (2/439)	
Delayed bleeding		0.510
Mucomuscular	0	
Conventional	0.5% (2/439)	
Postoperative hospital stay, median, days		< 0.001
Mucomuscular	1.9 ± 0.8	
Conventional	2.4 ± 1.7

**Table 2:** Risk factors of post C-ESD PEECS

	Patients with PEECS	Patients without PEECS	*P* value
Tumor size, median, mm	35.2 ± 15.2	30.9 ± 12.7	0.007
Operation time, median, min	72.8 ± 47.1	53.4 ± 30.8	< 0.001

### PEECS

A total of 74 cases of PEECS (9.7%) were observed in this study and was significantly higher in the conventional closure group than mucomuscular closure group (66/439 (15.0%) versus 8/325 (2.5%), *P* < 0.001). In the mucomuscular group, complete closure was achieved in 218 patients (218/325, 67.1%). The incidence of PEECS in complete and partial closure patients was comparable (2.3% versus 2.9% respectively, *P* = 0.999) (Table 3). All patients were conservatively managed with antibiotics and simple analgesics. The mean procedure time was longer in the PEECS cohort (72.8 versus 53.5 min, *P* < 0.001). The mean lesion size of patients with PEECS was 35.2 ± 15.2 mm, which was comparable to those without PEECS (30.9 ± 12.7 mm, *p* = 0.007) (Table 2).

**Table 3:** Clinical outcomes of partial closure versus complete closure in mucomuscular group

	Mucomuscular closure		*P* value
	Partial closure	Complete closure	
Case, *n*	105	218	–
Tumor size, median, mm	37.1 ± 15.3	27.4 ± 7.9	< 0.001
Operation time, median, min	62.7 ± 44.2	49.4 ± 24.3	< 0.001
Incidence of adverse events, percent (*n*)			
PEECS	3 (3/105,2.9%)	5 (5/218,2.3%)	0.999
Delayed perforation	0	0	–
Delayed bleeding	0	0	–
Postoperative hospital stay, median, days	2.0 ± 0.7	1.9 ± 1.3	0.172

### Severe adverse events

No SAEs or TTSC-related perforation occurred in the mucomuscular closure group. In the conventional closure group, intraoperative perforation occurred in one patient (0.2%) with rectal high-grade neoplasia that required conversion to laparoscopic Dixon surgery. Two patients (0.5%) had delayed perforation and received surgical repairment successfully without intestinal segment excision or colostomy. Both fully recovered after surgery. Two (0.5%) cases of delayed bleeding were successfully managed endoscopically without sequelae. The overall incidence of SAEs was not significantly different between the two groups (*P* = 0.999).

### Mucomuscular closure versus conventional closure

No significant differences were found in the demographic and lesion characteristics in both cohorts (Table 1). Procedure time was comparable between mucomuscular group and conventional group (53.7 min versus 56.5 min, *P* = 0.261). Mucomuscular closure group was associated with a shorter length of admission (1.9 days versus 2.4 days, *P* < 0.001).

### Risk factors for PEECS

Operation time ≥ 60 min (OR 1.8; 95% CI 1.1–3.1) and conventional closure method (OR 2.4; 95% CI 1.6–3.5) were significant independent predictive factors for PEECS on multivariate analyses (Table 4).

**Table 4:** Univariate and multivariate analysis of risk factors for post C-ESD PEECS

	Univariate analysis		Multivariate analysis	
	OR (95% CI)	*P* value	OR (95% CI)	*P* value
Age, years		0.794		
< 75	1			
≥ 75	1.115 (~0.493–2.522)			
Tumor size, mm		0.005		0.382
< 30	1		1	
≥ 30	2.065 (~1.247–3.419)		1.284 (~0.732–2.252)	
Operation time, min		< 0.001		0.029
< 60	1		1	
≥ 60	2.430 (~1.495–3.949)		1.830 (~1.065–3.144)	
Clip closure method		< 0.001		< 0.001
Mucomuscular	1		1	
Conventional	2.648 (~1.821–3.850)		2.386 (~1.631–3.490)	

## Discussion

PEECS is one of the most common adverse events of C-ESD, causing prolonged hospital stay and clinical manifestations such as abdominal pain and fever. Differentiation between PEECS and perforation can be challenging and sometimes result in unnecessary surgical exploration. Though the exact mechanism of PEECS has not been cleared, it is generally believed that reliable closure can reduce the incidence of PEECS [10, 11]. On the contrary, few studies suggested conventional closure of the C-ESD defect cannot decrease the incidence of adverse events or ameliorate inflammatory responses [12]. The negative outcome of endoscopic closure may result from unreliable closure of the defect. Inspired by surgical seromuscular suture, we proposed a mucomuscular closure method mimicking seromuscular suture, which can effectively reduce the tension of the intestinal wall. Our study showed that mucomuscular closure can effectively reduce the risk of PEECS and length of admission in C-ESD, thereby minimizing patient morbidity and utilization of limited healthcare resources.

This novel closure technique is safe and did not result in any cases of clip-related perforation. The incidence of delayed bleeding (0 versus 0.5%) and perforation (0 versus 0.5%) was lower in mucomuscular closure group even if not statistically significant. In addition, partial mucomuscular closure achieved comparable advantages in comparison with conventional TTSC closure in reduction of PEECS (2.9% versus 2.3%, *P* = 0.999) and length of hospital stay (2.0 days versus 1.9 days, *P* = 0.172).

Various methods of defect closure in C-ESD have been proposed in the last few years to improve perioperative safety during C-ESD [14–20]. The majority of these techniques focus on the alignment of the mucosal layer regardless of the MP layer. However, in cases with large defects or MP layer injury, complete mucosal closure with TTSC can be challenging and sometimes leaves a dead space between the mucosa and MP layer. Intestinal peristalses, edematous mucosa, and tissue exudation can easily result in premature dislodgement of clips and exposure of the defect.

The mucomuscular closure method is derived from the surgical technique of seromuscular suturing.

The colonic mucosa lacks the necessary thickness to uphold the integrity of the intestinal wall. Closure of the MP layer can provide sufficient support to ensure the tightness of TTSC and prevent early TTSC detachment as well as tiny leakage of the defect. Therefore, securing the muscularis propria layer is essential for achieving a durable closure and reducing PEECS.

Several studies have reported that reliable closure can reduce PEECS [9, 21, 22]. According to the previous literature reports, the incidence of PEECS after C-ESD varies, and few studies compared the incidence of PEECS among different suture methods. Our results showed that the incidence of PEECS was only 2.5% in mucomuscular closure group, which was significantly lower than that in the conventional closure group (15.0%, *P* < 0.001). Yamasaki et al. [10] compared line-assisted complete closure (LACC) with no closure after C-ESD regarding PEECS. The results showed that the incidence of PEECS was significantly lower in the LACC than in the control group (0% versus 12%, respectively; *P* = 0.030). Although the incidence of PEECS in LACC was down to zero, their sample size was small with 51 patients in each group. Conversely, Lee et al. did not demonstrate a significant reduction in the incidence of PEECS between conventional TTSC closure and no closure (8.2% versus 10.9%, respectively) [12]. The different conclusions drawn from the two studies may be because LACC is more robust and can eliminate dead space, whereas the conventional TTSC closure used by Lee fails to provide reliable suturing. No other studies compared PEECS specifically focusing on closure methods. Our research confirmed that mucomuscular closure can reduce the incidence of PEECS and other SAEs effectively. The results also supported that the modified closure method can provide solid defect closure.

Masunaga et al. [11] reported a comparable suturing technique termed the “Origami method” (OGM) with stringent requirements of complete closure and alignment of mucosal and MP layer. In our study, the incidence of PEECS and other adverse events in partial mucomuscular closure patients was comparable to that in complete closure patients, suggesting mucosal alignment and complete closure are nonessential regarding the reliability of the closure if adequate tension reduction is achieved (Table 3). Though there were no TTSC-related adverse events in our study, excessive pursuit of complete closure in large defects may lead to clip-related perforation, prolonged procedure time, and cost without additional benefit. In cases with large lesions, we recommend the direction of partial mucomuscular closure perpendicular to the long axis of the colon to achieve optimal tension reduction. Significant deep mural injury and intraoperative perforation area necessitate mucomuscular closure.

To our knowledge, we present here the largest prospective comparative study on endoscopic clip closure techniques in 764 patients. Mucomuscular closure is technically feasible, utilizing readily available TTSC, and can be widely adopted. Advanced techniques including endoscopic suturing may require additional cost, time, and expertise.

It is still indecisive whether to choose ESD or EMR when endoscopists encounter large colorectal lesions in consideration of perioperative safety [23]. One reason is that the incidence of PEECS as well as other perioperative adverse events is higher in C-ESD than in EMR cases [6, 24]. A variety of attempts such as underwater ESD and hybrid endoscopic resection have been reported to reduce the incidence of adverse events [25–27]. The higher incidence of PEECS in C-ESD cases may be attributed to larger lesion size, longer procedure time, and additional electrocauterization of the defect [9]. With a reliable closure technique, the incidence of PEECS in C-ESD was comparable to that in EMR [9, 10, 21, 22]. As we proposed the mucomuscular closure technique, the defect closure of ESD can be reliable and reassuring, which minimizes the risks and benefits the patients with en bloc resection.

Our study has a few limitations. First, there may be a correlation between the incidence of postoperative complications and endoscopist expertise. In our study, patients underwent conventional closure by multiple operators, but patients who underwent the modified mucomuscular closure were enrolled by three highly experienced operators. This discrepancy may introduce bias into our study results. Further studies involving general endoscopists and low-volume centers are needed to determine the learning curve of this technique and its generalizability. Second, the decision of partial or complete closure was determined by each endoscopist without predetermined criteria. Further detailed study is needed to identify lesions that can be managed by partial closure. Third, the visual analogue scale (VAS) was not introduced in our study due to the retrospective enrollment of the conventional cohort. The VAS score sensitively reflects patients’ pain intensity. At present, there is no research on the correlation between pain and the severity of PEECS. In future studies, incorporating VAS scores can provide a clearer understanding of the correlation between pain and the severity of PEECS and may potentially elucidate the mechanisms underlying PEECS.

Therefore, we recommend conducting large-scale, multicenter, prospective, randomized controlled trials in the future. Such studies could provide more comprehensive and reliable evidence regarding the safety and effectiveness of mucomuscular suturing method for colonic ESD.

## Conclusions

Mucomuscular closure method is more effective than conventional TTSC closure in reduction of post-C-ESD PEECS. Our study suggests that this novel closure method is both feasible and safe, potentially enhancing the appeal of C-ESD. It should be considered that the endoscopist’s experience could be a potential confounding factor influencing the study outcomes. Further research is necessary to obtain a more comprehensive understanding of this new method.

## Supplementary Information

Below is the link to the electronic supplementary material.

Supplementary file1 (MP4 7331 KB)

Supplementary file2 (DOCX 13 KB)

## Data Availability

No datasets were generated or analyzed during the current study.
