# Supplementary material for: Safety outcomes of mucomuscular closure versus conventional clip closure in ESD of large (> 15 mm) nonpedunculated colorectal polyps (LNPCPs)
Source: Tech Coloproctol. 2025 Dec 24;30(1):23. doi: 10.1007/s10151-025-03261-w (PMC12819494; doi:10.1007/s10151-025-03261-w)
Supplement: Supplementary file 2 — Supplementary file2 (DOCX 13 KB) [file 10151_2025_3261_MOESM2_ESM.docx]

Video 1. The defect of C-ESD was closed using mucomuscular closure method. A small full-thickness defect can be seen on the wound surface. Metal clips were used to directly suture the muscularis propria layer with gentle suction and complete closure was achieved.
